# The receipt of health information on neonatal dangers signs during the immediate postpartum period and its determinants in Ethiopia: a multilevel mixed-effects logistic regression analysis of the 2016 Ethiopian demographic health survey report

**DOI:** 10.1186/s12884-024-06605-w

**Published:** 2024-06-06

**Authors:** Aklilu Habte, Aiggan Tamene, Zablon Wale Sewalem

**Affiliations:** 1 School of Public Health, College of Medicine and Health Sciences, Wachemo University, Hosanna, Ethiopia; 2 Department of Clinical and Psychosocial Epidemiology, University of Groningen, Groningen, Netherlands

**Keywords:** Newborn danger signs, Health Information, Postpartum period, Determinants, Ethiopia

## Abstract

**Background:**

Identification of neonatal danger signs and immediate access to health care are two global efforts aimed at enhancing newborn and child survival by preventing 75% of neonatal deaths. Despite various small-scale studies on women’s awareness of neonatal danger signs in Ethiopia, little is known about the level of receiving health information on those danger signs during the immediate postpartum period at the national level. Hence, this study aimed at assessing the level, and its determinants of the service uptake in Ethiopia.

**Methods:**

The data for this study was taken from the Ethiopian Demographic and Health Survey (EDHS), which took place from January to June 2016 and covered all administrative regions of Ethiopia. A weighted sample of 7,589.8 women was analyzed using STATA version 16. To account for data clustering, a multivariable multilevel mixed-effect logistic regression analysis was employed to determine the effects of each predictor on the outcome variable. Adjusted odds ratio with its corresponding 95% confidence interval was used to declare the statistical significance of the explanatory variables.

**Results:**

The receipt of health information on neonatal danger signs during the immediate postpartum period was 10.70% [95% CI:10.01, 11.40]. Variables namely living in Metropolitans [AOR = 2.06; 95%CI: 1.48, 2.88] and Large central [AOR = 1.83; 95%CI: 1.38, 2.42] regions, being in the highest wealth quintile [AOR = 1.87; 95% CI: 1.23, 2.84], being nulliparous [AOR = 0.27; 95% CI: 0.08, 0.87] and primiparous[AOR = 0.61;95% CI: 0.46, 0.79], getting adequate antenatal visits [AOR = 2.42; 95% CI: 1.75, 3.33], institutional delivery [AOR = 5.91; 95% CI: 4.66, 7.53], and receipt of postnatal visits [AOR = 3.52; 95% CI: 2.84, 4.38] were identified as significant determinants of receiving health information on newborn danger signs.

**Conclusion:**

The findings revealed that unacceptably low uptake of health information on newborn danger signs during the immediate postpartum period in Ethiopia. A concerted effort is needed from all stakeholders in the health sector to enhance the uptake of maternal health services (antenatal care, skilled delivery service, and postnatal care). Healthcare providers should pay special attention to nulliparous and primiparous women during and after delivery, and the government should also focus on women of peripheral regions, who make up a large portion of the low coverage.

## Background

The neonatal period is the most critical period for child survival [1]. In 2019, 2.4 million children died in their first month of birth globally, with 6,500 neonatal deaths occurring on average every day. One-third of all newborn deaths occur within the first 24 h of life, and approximately three-quarters occur within the first week [2]. Five million children under the age of 5 died in 2020 from preventable or treatable causes, and the majority of these deaths were concentrated in sub-Saharan Africa and South Asia [3]. Approximately 98% of newborn deaths occur in developing nations, with a global neonatal mortality rate of 17 deaths per 1000 live births in 2019 [2, 4]. The neonatal mortality rate (NMR) was highest in Sub-Saharan Africa and with 27 deaths per 1000 live births [5]. The figure is also has remained unacceptably high in Ethiopia (33 in 1000 Live births) for the past 20 years despite a significant global decline [6, 7].

The main causes behind neonatal death are premature birth and its complications, pneumonia, birth asphyxia, congenital anomalies, diarrhea, and malaria [8]. Almost all of these causes are preventable or treatable through immunization, adequate nutrition, clean water, and care provided by a well-trained healthcare provider [9]. Identification of Newborn Danger Signs (NDSs) and prompt medical care seeking are two global strategies designed by World Health Organization (WHO) and United Nations Children’s Fund (UNICEF) to improve the survival of newborns [2, 10]. These signs signal a high risk of neonatal morbidity and mortality and need to be quickly identified by mothers and caregivers, for immediate therapeutic intervention [11, 12]. UNICEF and WHO define the following signs and symptoms as danger signs in newborns: fever, feeding problems, convulsions, fast breathing, abnormal body movement, yellow soles, reddened or pus-draining umbilicus, and reddened or pus-draining eyes [8, 13].

Reducing neonatal deaths requires improving healthcare-seeking behavior through immediate diagnosis of fatal illnesses, and good health information on NDSs [14, 15]. Newborns who experienced danger signs were twice as likely to die as compared to their counterparts [16]. As a result, mothers need to be counseled to recognize danger signs in order to effectively identify newborns at risk of morbidity and mortality and obtain appropriate health care [17].

According to scientific evidence, a variety of factors were influencing the adoption of health information on NDSs. Higher educational attainment of mothers and husbands, proximity to medical facilities, familiarity with neonatal danger signs before pregnancy, receipt of ANC and PNC services, and access to mass media were all noted as potential predictors of service uptake [14, 18–20].

Immediate detection of NDSs is crucial for seeking prompt treatment for newborn ailments, particularly in resource-limited settings such as Ethiopia. Simple and low-cost interventions, such as informing mothers about NDSs and empowering them to seek health care, are estimated to prevent 75% of neonatal deaths [21]. Although maternal knowledge of NDSs is critical for newborn and child survival, evidence from around the world [22], including Ethiopia [23], indicates that mothers’ knowledge in this area is still low, necessitating intensive health information during the prenatal and postpartum periods.

Despite numerous small-scale studies on women’s knowledge of NDSs in Ethiopia [14, 24–27], little is known about the status and determinants of receiving health information on NDSs during the immediate postpartum period(PPP) at the national level. Thus the actual figure of health information about NDSs needs to be known at the national level to take appropriate interventions at service delivery points. Hence, this study aimed at assessing the receipt of health information on NDSs, and its predictors in Ethiopia by using the 2016 EDHS report. Assessing and identifying the level and determinants would alert policymakers and healthcare providers to plan for resources and pay special attention to reducing neonatal mortality and morbidity, which may eventually contribute to the struggle to meet national targets.

## Methods

### Data source, study period, and design

The data used for this study was extracted from the child (KR) file of the 2016 EDHS report, a population-based, nationally representative data conducted by the Central Statistical Agency (CSA) in collaboration with the Ministry of Health through the DHS Program. The survey was conducted from January 18 to June 27, 2016 [28]. Data were obtained from their URL: www.dhsprogram.com by contacting them via personal email communication with a possible justification for the data request. All nine regional states (Afar, Amhara, Benishangul-Gumuz, Gambela, Harari, Oromia, Somali, Southern Nations, Nationalities, and People’s Region, and Tigray), and the two administrative cities (Addis Ababa and Dire-Dawa) of Ethiopia were included in the survey.

### The population of the study

The source population was all women with kids, and who gave birth in the last 2 years preceding the survey. Of the 10,641 mothers with children in the Kid’s Record (KR) file, only 7,193 had complete information on receiving health information on NDSs and they represented the study population. Thus, the analyses and the findings were based on the records of 7,193 study participants (a weighted sample of 7,589.8).

### Sampling procedures

The survey employed a stratified two-stage cluster sampling technique using the 2007 population and housing census to select respondents. Sampling strata were formed by dividing each of the regions included in the survey into urban and rural settings. The first step was to select 645 clusters (202 urban and 443 rural areas) with a probability proportional to the size of the enumeration area and independent selection within each stratum. In all of the selected Enumeration areas, the household listing was completed between September and December 2015. The second stage involved the selection of 28 households per cluster using an equal probability systematic selection of eligible women with children. A total weighted sample of 7,589.8 children with mothers were eligible for being included in the analyses. Furthermore, the survey design and methodology were detailed in the 2016 EDHS [28].

### Measurement of variables of the study

Outcome variables: The outcome of interest in this study was the receipt of health information on neonatal danger signs, which was measured as a categorical variable as receiving or not receiving the service. It was assessed by a question “Did a healthcare provoder/s inform you of neonatal danger signs during the first two days of your childbirth?”. This variable was dichotomized into “No = 0” (for women who reported they didn’t get any health information on NDSs and who responded as ‘I don’t know’) and “Yes = 1”(for women who reported they got the information on at least one NDSs).

### Explanatory variables

Based on a review of the recent literature, potential factors associated with service uptake were extracted from the dataset and classified into individual and community-level factors (Table 1).

**Table 1:** List of the individual- and community-level factors that affect the uptake of health information on NDSs in immediate PPP in Ethiopia, 2016

Individual level variables	Socio-demographic and economic factors	Religion, ethnicity, age of women, education, and occupation of women and partners, place of residence (urban and rural), wealth index, and family size
	Obstetric factors	Parity, gravidity, total number of births in the last five years, pregnancy status when she became pregnant (wanted, mistimed, unwanted), total children ever born, and ever had a termination of pregnancy)
	Health service-related characteristics	Frequency of Antenatal care visits, place of receiving ANC, contraceptive use, decision-making power over own health care (self-decision/joint decision with husband, husband alone, and other),
	Media exposure	Exposure to the newspaper, radio, and television (not at all, less than once a week, or at least once a week),
	Difficulty in accessing healthcare	Distance to a health facility, obtaining permission to visit a health facility, and obtaining the money required for treatment
Community-level factors	Geographical regions and place of residence	The geographical regions (small peripheral, larger central, or metropolitan) are based on their geopolitical features [32], and residence(Urban or rural). Small peripherals include Afar, Somali, Benishangul, and Gambella regions. The larger central regions include Tigray, Amhara, Oromia, and Sothern Nations Nationalities and Peoples Region (SNNPRs), while the Metropolitan includes Harari region, Dire Dawa, and Addis Ababa administrative cities [32, 33].

Wealth index: is a composite measure of a household’s cumulative living standard that is generated using simple data on a household’s ownership of certain assets such as televisions, bicycles, and cars; dwelling characteristics such as flooring material; type of drinking water source; and toilet and sanitation facilities [21]. Using principal component analysis, households were allocated scores depending on the type and amount of the aforementioned assets and household items they owned. Finally, each household was given a continuous asset score, and they were divided into five wealth quintiles [28].

### Data management and analysis

The sample allocation to different regions, as well as urban and rural settings, was not proportional in the EDHS. As a result, sample weights were used to estimate proportions and frequencies to account for disproportionate sampling and non-response. The weighting procedure was thoroughly explained in the 2016 EDHS report [28]. Before analysis, data were checked for missing values and data cleaning, variable recoding, labeling, categorization, and re-categorization were done. STATA/SE version 16.0 was used for the analysis. Both descriptive statistics, such as frequencies and proportions, and analytic statistics were computed. A weighted analysis was performed to account for the unequal probability of selection between strata due to the non-proportional allocation of samples to different regions, places of residence, and non-response rate among study participants [28]. The variance inflation factor (VIF) with a cut-off value of 10 was used to test for the presence of multicollinearity among independent variables. However, the VIF value for all predictors was less than 10, indicating that there was no inter-variable multicollinearity.

To estimate both the independent (fixed) effect of the explanatory variables and the community-level (random) effect on our dependent variable, a two-level mixed-effect logistic regression model was fitted. First, to examine the relationship between each predictor and the outcome variable, a bivariable multilevel logistic regression analysis was performed. In this analysis, variables with *p*-values less than 0.25 were considered candidates for multivariable logistic regression analysis. To account for data clustering, a multivariable multilevel mixed-effect logistic regression analysis was run to determine the effects of each predictor on the receipt of health information on NDSs. In a multivariable multilevel mixed-effect logistic analysis, four models with the variables of interest were fitted, and the best-fitting model was chosen. Model-I is a null model, Model II is a model with only individual-level factors, Model III is a model with only community-level factors, and Model IV is a full model. The full model (Model IV) was fitted to examine the effect of individual and community-level predictors on the outcome variable at the same time. The adjusted odds ratio with the corresponding 95% confidence interval was computed and reported to demonstrate the strength of the association and its significance. Variables having a *p*-value < 0.05 were considered as having a significant association with the outcome variable. The model comparison was done using deviance and the fourth model with the lowest deviance (4022.80) was selected as the best-fitted model (Table 2).

**Table 2:** Random intercept variances and model fit statistics comparison of two-level mixed-effect logistic regression model predicting the receipt of health information on NDSs during immediate PPP in Ethiopia, 2016

Measures	Model-I (null model)	Model-II (individual factors)	Model-III (community factors)	Model-IV (full model)
Variance	2.07	0.51	1.12	0.47
ICC	0.39	0.13	0.25	0.12
AIC	4916.49	4107.25	4714.33	4090.81
BIC	4930.26	4320.56	4748.74	4324.76
MOR	3.94	1.98	2.76	1.93
PCV	Reference	0.75	0.46	0.77
**Model fitness**				
Log-likelihood	-2456.25	-2033.63	-2352.17	-2011.40
Deviance	4912.5	4067.26	4704.34	4022.8

## Results

### Sociodemographic characteristics of women

This study included a total of 7,589.8 weighted women who responded to a question if they were counseled on neonatal danger signs within two days following birth within the five years preceding the survey. The mean (± SD) age of study participants was 29.25(± 6.84) years, of which more than a quarter (28.53%) of them belongs to the age group 25–29 years. the majority, 6,899.5 (90.9%) of the respondents were from larger central regions. The vast majority of respondents, 6,620.9(87.2%) were rural residents. Nearly two-thirds (63.1%) of the respondents had no formal education whereas 2,149.6(28.3%) of respondents attended primary education (Table 3).

**Table 3:** Sociodemographic characteristics of women in the reproductive age group in Ethiopia, EDHS 2016

Variable categories	Weighted frequency	Percent
**Regions**		
Large central regions ^a^	6,899.5	90.9
Small peripheral regions ^b^	441.3	5.8
Metropolitans ^**c**^	249.0	3.3
**Current Age**		
15–19	338.9	4.5
20–24	1,465.0	19.3
25–29	2,165.3	28.5
30–34	1,661.1	21.9
35–39	1,206.1	15.9
40–44	546.4	7.2
45–49	206.8	2.7
**Current Marital status**		
In marital relation	7,020.1	92.5
Live with partner	88.48	1.2
Not in marital relation	481.1	6.3
**Religion**		
Orthodox	2,882.1	38.0
Muslim	2,824.0	37.2
Protestant	1,651.4	21.8
Traditional	96.7	1.3
Catholic	71.5	0.9
Other	64.0	0.8
**Educational status**		
No education	4,791.0	63.1
Primary	2,149.6	28.3
Secondary	419.6	5.5
Higher	229.5	3.0
**Husband’s education**		
No education	3,345.8	47.1
Primary	2,731.3	38.4
Secondary	612.8	8.6
Higher	375.8	5.3
Don’t know	42.9	0.6
**Occupation**		
Unemployed	4,078.1	53.7
Employed	3,511.6	46.3
**Residence**		
Urban	968.8	12.8
Rural	6,620.9	87.2
**Family size**		
<=5memeber	3,636.3	47.9
> 5	3,953.4	52.1
**Wealth index combined**		
Poorest	1,651.4	21.7
Poorer	1,654.1	21.8
Middle	1,588.2	20.9
Richer	1,426.7	18.8
Richest	1,269.3	16.7

^*a*^ *Tigray, Amhara, Oromia, South Nation Nationalities*

^*b*^ *Somali, Afar, Gambela, Benshangul Gumuz*

^*c*^ *Addis Ababa, Dire Dawa, Harar*

### Obstetric characteristics of the respondents

The majority of respondents 3,477.8(45.8%) were multiparous (having 2–4 living children), followed by grand multiparous (having five or more living children) 2,526.8 (33.3%). Nearly three-quarters (73.4%) of women reported that their pregnancy was planned, and 680.2 (9.0%) experienced termination of pregnancy. More than half (51.52%) of respondents got at least four antenatal visits. Two-thirds, 5,066.3(66.7%) of deliveries were taken place at home and only 632.2(8.3%) of women received postnatal check-ups within 2 months of delivery. The majority, 273.4(43.3%) of women got their postpartum visit by nurses (Table 4).

**Table 4:** Obstetric characteristics of women in the reproductive age group in Ethiopia, EDHS 2016

Variable categories	Weighted frequency	Percent
**Parity**		
Nulliparous	49.0	0.7
Primiparous	1,536.1	20.2
Multiparous	3,477.8	45.8
Grand multiparous	2,526.8	33.3
**Pregnancy status when she became pregnant**		
Wanted	5,573.5	73.4
Mistimed	1,321.2	17.4
Unwanted	695.1	9.2
**Births in the last five years**		
One	4,631.5	61.0
Two	2,521.8	33.2
More than two	436.5	5.8
**Total children ever born**		
One	1,434.5	18.9
2–5	4,033.0	53.1
>=6	2,122.3	28.0
Ever had a termination of pregnancy		
No	6,909.5	91.0
Yes	680.2	9.0
Frequency of ANC		
No visit	2,833.2	37.3
1 visit	334.5	4.4
2–3 visits	2,007.4	26.4
≥ 4 visits	2,414.6	31.8
**Place of delivery**		
Home delivery	5,066.3	66.7
Facility delivery	2,523.5	33.3
**Sex of child**		
Male	3,940.4	51.9
Female	3,649.3	48.1
**Delivery by Caesarean section**		
No	7,406.5	97.6
Yes	183.3	2.4
**Received postnatal check-up within 2 months**		
Yes	632.2	8.3
No	6,957.5	91.7
**A person who performed a postnatal check-up**		
Doctor	71.9	11.4
Nurse	273.4	43.3
Midwife	52.0	8.4
Health officer	12.9	2.0
Health extension work	208.2	32.8
Traditional birth attendants	13.5	2.1
**When a child put to the breast**		
Immediately after birth	5,265.5	69.4
< 1 h	214.9	2.8
At first hour	614.7	8.1
> 1 h.	1,494.6	19.7

### Individual and health system-related characteristics of respondents

The majority of respondents had no media exposure, with only 135.3(1.8%), 1,069.3(14.1%), and 724.2(9.5%) of women reporting reading a newspaper or magazine, listening to the radio, and watching television at least once a week, respectively. In terms of decision-making power in seeking health care, only 13.0% of women decided independently, whereas 62.2% of women reported that they made a joint decision to seek health care. Distance to health facilities and obtaining permission to seek medical care were big problems for 4,406.4 (58.1%) and 2,764.5 (36.4%), respectively. Only 317.3(4.2%) of women were enrolled in health insurance(Table 5).

**Table 5:** Individual characteristics of women in the reproductive age group in Ethiopia, EDHS 2016

Variables	Weighted frequency	Percent
**Reading newspaper**		
Not at all	7,050.1	92.9
Less than once a week	404.3	5.3
At least once a week	135.3	1.8
**Listening to a radio**		
Not at all	5,490.9	72.3
Less than once a week	1,029.6	13.6
At least once a week	1,069.3	14.1
**Watching television**		
Not at all	6,101.7	80.4
Less than once a week	763.9	10.1
At least once a week	724.2	9.5
**Had a mobile phone**		
No	6,216.5	81.9
Yes	1,373.2	18.1
**Ever taken a drink that contains alcohol?**		
No	5,115.5	67.4
Yes	2,474.2	32.6
**Who decides on healthcare?**		
Respondent alone	984.5	13.0
Respondent and husband (joint decision)	4,720.0	62.2
Others	1,885.2	24.8
**Distance to a health facility**		
Big problem	4,406.4	58.1
Not a big problem	3,183.3	41.9
**Getting permission to go to a health facility**		
Big problem	2,764.5	36.4
Not a big problem	4,825.3	63.6
**Getting money needed for treatment**		
Big problem	4,546.9	59.9
Not a big problem	3,042.8	40.1
**Covered by health insurance**		
No	7,272.5	95.8
Yes	317.3	4.2

### The receipt of health information on NDSs by respondents

**Fig. 1:**
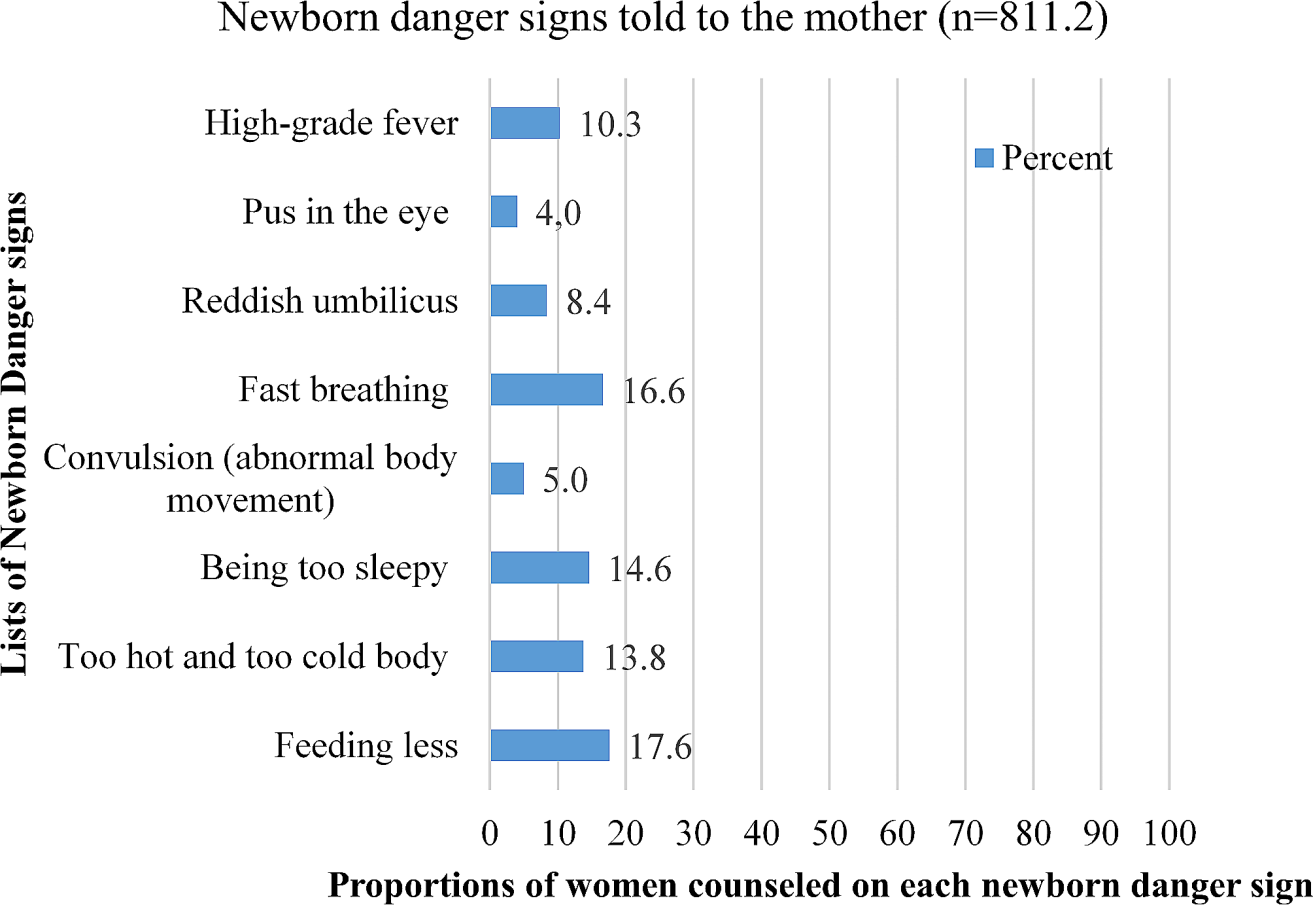
Percentage of women informed about each neonatal danger sign during the immediate postpartum period in Ethiopia, EDHS 2016

Accordingly, only 811.2, 10.70% [95% CI:10.01, 11.40] of women, got health information on at least one newborn danger sign during the immediate postpartum period. The 2016 EDHS collected data on health information on the eight newborn danger signs. Those danger signs were feeding problems (feeding less), too hot and too cold body parts, being too sleepy, abnormal body movement (convulsion), fast breathing, reddish umbilicus, presence of pus in the eye, and High-grade fever. The commonest newborn danger sign told to the women was feeding problems (43.5%) the sign of feeling sleepy (35.9%) (Fig. 1).

### Model building and selection

Model IV, a model adjusted for both individual and community-level factors, had the lowest AIC value and was selected as the best model fit for the data. Furthermore, as fitted models progressed from the empty model (Model-I) to Model-II, Model-III, and Model-IV, the value of the log-likelihood results consistently decreased, indicating that the fitted models were a better fit to the data. Intraclass correlation coefficient (ICC), a proportional change in variance (PCV), and median odds ratio (MOR) were used to estimate the random effect results. The result of the random effects model showed that the variance of the random factor in the null model was 1.12 [95% CI: 0.87, 1.46], indicating the existence of variation in the receipt of health information on NDSs. Thus, to account for this variation, a multilevel logistic regression model was considered for further analysis.

The random effect model resulted in an ICC of 0.39, indicating that 39% of the variation in NDSs health information received by women during PPP in Ethiopia could be attributed to EA differences. Furthermore, MOR indicated an unexplained community variation in the receipt of health information on NDSs from 3.94 (null model) to 1.93 (full model), indicating that when all predictors are considered, the effect of clustering is still statistically significant. On the other hand, more than three-quarters (77.0%) of the unexplained variation in the uptake of health information could be attributable to the unobserved community‑ and household‑level factors together (Table 2).

### Factors affecting the receipt of health information on NDSs: a bivariable and multivariable multilevel mixed‑effects logistic regression analyses

In the bivariable multilevel logistic regression, region, residence, respondents’ educational status, being employed, wealth index, parity, frequency of ANC, place of delivery, mode of delivery, receiving a postpartum visit, exposure to media (newspaper, radio, and television), having a mobile phone, distance to a health facility, having permission to seek medical care, having money for medical care, and enrollment in health insurance were associated with the receipt of health information on NDSs(*p*-value < 0.25) (Table 6).

**Table 6:** Results of a bivariable multilevel logistic regression analysis to identify the factors affecting the receipt of health information on NDSs during immediate PPP in Ethiopia, 2016

Variable categories	Received health information on NDSs		COR(95% CI)	*p*-value
	Yes (%)	No (%)		
**Educational status**				
Higher	56.6 (7.0)	172.9(2.5)	3.93(2.79, 5.53)	< 0.001
Secondary	111.9(13.8)	307.7(4.5)	3.25(2.47, 4.27)	< 0.001
Primary	299.8 (36.9)	1,849.8(27.4)	2.10( 1.73, 2.54)	< 0.001
No education	342.8(42.3)	4,448.2(65.6)	1	
**Occupation**				
Employed	455.8 (56.2)	3,055.8(45.1)	1.53(1.28, 1.81)	< 0.001
Unemployed	355.4 (43.8)	3,722.7(54.9)	1	
**Wealth index combined**				
Richest	316.7 (39.1)	952.6(14.0)	8.83 (6.58, 11.85)	< 0.001
Richer	174.9 (21.6)	1,251.8(18.5)	4.12 (2.98, 5.67)	< 0.001
Middle	136.6 (16.8)	1,451.6(21.4)	2.66 (1.93, 3.68)	< 0.001
Poorer	103.5(12.7)	1,550.5(22.9)	1.98 (1.44, 2.73)	< 0.001
Poorest	79.4(9.8)	1,571.9(23.2)	1	
**Parity**				
Nulliparous	2.3(0.3)	46.7(0.7)	0.52(0.17, 1.61)	0.259
Primiparous	219.4( 27.0)	1,316.6(19.4)	1.35(1.07, 1.71)	0.012
Multiparous	398.2(49.09)	3,079.5(45.4)	1.16(0.95, 1.42)	0.141
Grand multiparous	191.2(23.6)	2,335.6(34.46)	1	
**Frequency of ANC**				
≥ 4 visits	500.9(61.7)	1,913.7(28.2)	8.85(6.58,11.91)	< 0.001
2–3 visits	213.0(26.3)	1,794.4(26.5)	4.59( 3.36, 6.26)	< 0.001
One visit	24.4(3.0)	310.1(4.6)	3.22( 1.97, 5.28)	< 0.001
No visit	72.9( 9.0)	2,760.3(40.7)	1	
**Place of delivery**				
Facility delivery	644.0(79.4)	1,879.5(27.7)	10.91(8.83, 13.5)	< 0.001
Home delivery	167.2(20.6)	4,899.0(72.3)	1	
**Sex of child**				
Male	448.2(55.3)	3,492.2(51.5)	1.08(0.92, 1.27)	0.331
Female	363.0(44.7)	3,286.3(48.5)	1	
**Delivery by Caesarean section**				
Yes	72.5 ( 8.94)	110.7(1.6)	0.24(0.17, 0.33)	< 0.001
No	738.7(91.1)	6,667.8(98.4)	1	
**Received postnatal check-up**				
Yes	217.8( 26.8)	414.4(6.1)	4.76(3.95, 6.80)	< 0.001
No	593.4(73.2)	6,364.1(93.9)	1	
**Autonomy in healthcare utilization**				
Self-decision	150.0( 18.5)	834.47(12.3)	1.19(0.92, 1.54)	0.291
Joint decision with a husband	493.2(60.8)	4,226.7(62.3)	1.11(0.90, 1.36)	0.324
By others	167.9( 20.7)	1,717.3(25.4)	1	
**Reading newspaper**				
At least once a week	30.5( 3.8)	104.7(1.5)	2.61(2.00, 3.42)	< 0.001
Less than once a week	121.1(14.9)	283.2(4.2)	1.93(1.22, 3.04)	0.004
Not at all	659.5( 81.3)	6,390.5(94.3)	1	
**Listening radio**				
At least once a week	180.6( 22.3)	888.6(13.1)	2.45(1.97, 3.06)	< 0.001
Less than once a week	163.5(20.1)	866.1(12.8)	1.70(1.36, 2.14)	< 0.001
Not at all	467.1(57.6)	5,023.8(74.1)	1	
**Watching television**				
At least once a week	196.8( 24.3)	527.4(7.8)	3.55(2.81, 4.49)	< 0.013
Less than once a week	114.3(14.1)	649.6(9.6)	1.75(1.33, 2.30)	0.021
Not at all	500.1(61.6)	5,601.5(82.6)	1	
**Had a mobile phone**				
Yes	307.5(37.9)	1,065.7(15.7)	2.97(2.45, 3.61)	0.002
No	503.7(62.1)	5,712.8 (84.3)	1	
**Distance to a health facility**				
Big problem	323.4(39.9)	4,083.1(60.2)	0.56(0.46, 0.68)	0.001
Not a big problem	487.8(60.1)	2,695.4(39.8)	1	
**Getting permission to go to a health facility**				
Big problem	177.3(21.9)	2,587.1(38.2)	0.67(0.54, 0.83)	0.012
Not a big problem	633.9(78.1)	4,191.4(61.8)	1	
**Getting money needed for treatment**				
Big problem	384.6(47.4)	4,162.3(61.4)	0.66(0.55, 0.78)	0.003
Not a big problem	426.6( 52.6)	2,616.2(38.6)	1	
**Covered by health insurance**				
Yes	52.4( 6.5)	264.8(3.9)	1.86(1.26, 2.76)	0.002
No	758.8( 93.5)	6,513.7(96.1)	1	
**Region**				
Metropolitans	90.8(11.2)	158.2(2.3)	8.90(6.13, 12.92)	< 0.001
Large central regions	699.1( 86.2)	6,200.4(91.5)	3.06(2.22, 4.23)	< 0.001
Small peripheral regions	21.3( 2.6)	419.9(6.19)	1	
**Residence**				
Urban	260.7( 32.1)	708.1(10.4)	5.18(3.95, 6.80)	< 0.001
Rural	550.5(67.9)	6,070.4(89.6)	1	

**Key**: 1: Reference category; COR = Crude odds ratio, ^**^ Statistically significant at *p*-value < 0.25

A multivariable multilevel binary logistic regression analysis was run. After adjusting for individual and community-level variables in the final model (Model-IV), region, wealth index, parity, frequency of ANC, place of delivery, mode of delivery by cesarean section, and receipt of postnatal visits, were significantly associated with the uptake of health information on NDSs (*p*-value < 0.05).

Accordingly, the odds of receiving health information on NDSs were 1.87 (AOR = 1.87; 95% CI: 1.23, 2.84) times and 1.74 (AOR = 1.74; 95% CI: 1.24, 2.45) times higher among women from the richest and richer wealth index categories, respectively, as compared with women from the poorest category. Nulliparous and primiparous women were 63% (AOR = 0.27; 95% CI: 0.083, 0.87) and 39% (AOR = 0.61; 95% CI: 0.46, 0.79) less likely to get health information on NDSs as compared to grand multiparous, respectively. The frequency of ANC visits was also found to be a significant predictor of receiving health information NDSs. Women who had four or more ANC visits had a 2.42 times higher chance of receiving NDSs health information than women who had no ANC visits (AOR = 2.42; 95% CI: 1.75, 3.33). The place and mode of delivery were identified as significant predictors of receiving health information on NDSs. Women who gave birth in a health facility had a 6 times greater chance of receiving NDSs health information than women who gave birth at home (AOR = 5.91; 95% CI: 4.66, 7.53). Women who gave birth via cesarean section, on the other hand, were 44% less likely to be counseled on NDSS than women who gave birth via spontaneous vaginal delivery (SVD) (AOR = 0.54; 95% CI: 0.39, 0.74). The odds of being counseled on NDSs were 3.52 times higher among mothers who received a postnatal checkup compared to those who did not (AOR = 3.55; 95% CI: 2.84, 4.38) (Table 7).

**Table 7:** Results of a multilevel multivariable logistic regression analysis to identify the factors affecting the uptake of health information on NDSs during immediate PPP in Ethiopia, 2016

Variable categories	Model-I (null model)	Model II (individual-level factors)	Model III (community-level factors)	Model-IV (full model)
	AOR (95% CI)	AOR (95% CI)	AOR (95% CI)	AOR(95% CI)
**Educational status**				
Higher		1.73(1.49, 2.28)		0.87(0.58, 1.31)
Secondary		0.89(0.66, 1.23)		1.04(0.75, 1.45)
Primary		1.07(0.87, 1.32)		1.16(0.93, 1.45)
No education		1		1
**Women’s occupation**				
Employed		1.35(1.13, 1.61)		1.30(0.96, 1.55)
Unemployed		1		1
**Wealth index combined**				
Richest		2.21(1.45, 3.03)^*^		1.87(1.23, 2.84)^*^
Richer		2.03(1.45, 2.84)^*^		1.74(1.24, 2.45) ^*^
Middle		1.55(1.11, 2.17)^*^		1.34(0.96, 1.89)
Poorer		1.25(0.90, 1.75)		1.11(0.79, 1.56)
Poorest		1		1
**Parity**				
Nulliparous		0.27(0.08, 0.85)^*^		0.27(0.083, 0.87) ^*^
Primiparous		0.62(0.47, 0.82)^*^		0.61(0.46, 0.79) ^*^
Multiparous		0.81(0.65, 1.21)		0.81(0.64, 1.12) ^*^
Grand multiparous		1		
**Frequency of ANC**				
≥ 4 visits		2.57(1.79, 3.40)^*^		2.42(1.75, 3.33) ^*^
2–3 visits		1.96(1.40, 2.72)^*^		1.92(1.38, 2.67) ^*^
One visit		1.76(1.04, 2.98)^*^		1.72(1.02, 2.92) ^*^
No visit		1		1
**Place of delivery**				
Facility delivery		6.26(4.94, 7.94)		5.91(4.66, 7.53)^*^
Home delivery		1		1
**Delivery by caesarean section**				
Yes		0.51(0.37, 0.70)^*^		0.54(0.39, 0.74)^*^
No		1		1
**Received postnatal check-up**				
Yes		3.46(2.78, 4.31)		3.52(2.84, 4.38)^*^
No		1		1
**Reading newspaper**				
At least once a week		1.28(0.96, 1.71)		1.01(0.63, 1.63)
Less than once a week		1.02(0.63, 1.63)		1.24(0.93, 1.65)
Not at all		1		1
**Listening radio**				
At least once a week		1.41(1.11, 1.80)		1.35(0.96, 1.72)
Less than once a week		1.15(0.90, 1.47)		1.11(0.87, 1.42)
Not at all		1		1
**Watching television**				
At least once a week		0.78(0.57, 2.06)		0.84(0.62, 1.15)
Less than once a week		0.87(0.64, 1.17)		0.76(0.55, 1.23)
Not at all		1		1
**Had a mobile phone**				
Yes		1.16(0.91, 1.49)		1.25(0.97, 1.59)
No		1		1
**Distance to a health facility**				
Big problem		1.05(0.84, 1.31)		1.03(0.83, 1.29)
Not a big problem		1		1
**Getting permission to go to a health facility**				
Big problem		0.87(0.68, 1.12)		0.87(0.68, 1.11)
Not a big problem		1		1
**Getting money needed for treatment**				
Big problem		1.11(0.90, 1.37)		1.11(0.90, 1.37)
Not a big problem		1		1
**Covered by health insurance**				
Yes		1.22(0.83, 1.79)		1.13(0.77, 1.65)
No				1
**Region**				
Metropolitans			3.87(2.65, 5.65)	2.06(1.48, 2.88)^*^
Large central regions			3.21(2.37, 4.34)	1.83(1.38, 2.42)^*^
Small peripheral regions			1	
**Residence**				
Urban			3.93(2.90, 5.32)	0.89(0.62, 1.29)
Rural			1	1

**Key**: 1: Reference category; AOR = Adjusted odds ratio, COR = Crude odds ratio, ^**^ Statistically significant at *p*-value < 0.05

## Discussion

This study aimed at assessing the magnitude and determinants of receiving health information on NDSs among postpartum women in Ethiopia by using the EDHS report of 2016. Accordingly, the receipt of health information on NDSs among postpartum mothers was 10.70%. In a multivariable multilevel mixed-effect model, variables namely region, wealth index, parity, frequency of ANC, place of delivery, mode of delivery by cesarean section, and receipt of postnatal visits were significantly associated with the receipt of health information on NDSs.

The magnitude of receipt of health information on NDSs in the current study was found to be low as compared to studies conducted in China (42.%) [29], Kenya (51.8%) [30], and 42.8% [31] ), Southern Ethiopia (35.5%) [25], West-central Ethiopia (65.5%) [32], and northwest Ethiopia(64.5%) [33]. All these disparities might be due to variations in the sociodemographic characteristics of study participants, and health-system-related characteristics (like shortage of healthcare providers and distance to health facilities). Furthermore, the difference could also be due to differences in study participants (the current finding was focused on the uptake of health information in the immediate PPP), study setting, and time. The uptake was inadequate in comparison to the UNICEF standard that every woman be counseled about those danger signs during her pregnancy and PPP [2, 10]. Furthermore, this report implies that thousands of mothers did not receive health information on NDSs during immediate PPP, and thus the Ministry of Health, healthcare providers, and community health workers must play an important role in raising awareness of this issue in order to prevent neonatal mortality and morbidity.

Regional variations were attributed to a significant difference in the receipt of health information on NDSS were attributed to a significant difference. This was supported by a study done in Ethiopia [34], and Nigeria [35]. This could be because of a lack of adequate transportation infrastructure, socioeconomic discrepancies, educational status, and limited health facilities in these places, making it difficult for health information to reach these women. Furthermore, disparities in health system capacity, especially the availability of qualified healthcare personnel, might have an impact on health information communication. Communities in regions where competent health providers are few may struggle to receive comprehensive and timely information about newborn danger signs.

The odds of receiving health information on NDSs were higher among women from the richest and richer wealth index categories, as compared with women from the poorest category. Studies conducted in Bangladesh [36], Papua New Guinea [37], and India [38] supported this finding, that women in a higher wealth quintile had a good uptake of maternal and child health services. This could be because women from higher economic classes have more money to visit health facilities for prenatal and intrapartum care [39, 40]. On the other hand, women who belong to the richest household usually have higher educational status [40, 41], access to mass media, and the ability to spend more money to take frequent ANC visits in which they got adequate health information about NDSs. Although maternal health services are currently free at the facility level in Ethiopia, the majority of the expenses on the way to the health facility, such as transportation and food, are backed by the clients, making access to maternal and child health services challenging.

The likelihood of receiving health information on NDSs was lower among nulliparous and primiparous women as compared to grand multiparous, respectively. Some findings also reported that some breast problems, such as breast pain and infections, are common in primiparous women and are known to delay the receipt of health information on NDSs [37]. In addition, women who have had no or limited childbirth experience may have poor knowledge of the various risks and issues that can develop during the immediate stages of their baby’s life, as well as the value of health information during PPP [42]. This finding also suggests that health promotion interventions and health communication initiatives should target first-time and/or young mothers.

The analysis indicates that there was a significant positive association between the uptake of adequate ANC visits and health information on NDSs. This finding was supported by DHS data analysis of Middle-income countries [43], Madagascar [44], Kenya [30, 45], Uganda [46], Nigeria [43], Ethiopia [47–50]. This could be because as the number of ANC visits increases, so does the likelihood that women gave birth at health facilities, which increases the uptake of health information on NDSs. Furthermore, women who receive adequate ANC are more likely to have frequent interactions with healthcare personnel, develop to feel at ease with the staff, and have greater access to being informed about the NDSs during the immediate PPP.

Similarly, the uptake of postnatal care is also identified as a significant predictor of receiving health information on NDSs, in tandem with studies conducted in China [29], Kenya [51], Ghana [52], and Ethiopia [19, 26, 48, 53]. One possible explanation is that mothers who received postnatal care may have received health education about NDSs from healthcare providers, which is one of the essential components of postpartum visits [54]. Furthermore, postpartum care health information is a provision of adequate health information on the immediate identification of maternal and NDSs after birth, as well as improving maternal health-seeking behavior.

The odds of receiving health information on NDSs were higher among women who gave birth at health facilities and this was supported by studies conducted in Nigeria [55], Ghana [56], Uganda [46], and Ethiopia [49]. This could be because a Skilled delivery service frequently involves the presence of trained healthcare professionals, such as doctors, nurses, or midwives, who have knowledge and competence in NDSs along with better communication skills, which may increase the likelihood of receiving the service. In addition, mothers who gave birth in health facilities were more likely to be visited by healthcare providers for immediate postpartum care and during this moment they might be counseled about NDSs.

The analysis was based on nationally representative data with a large sample size, which was collected using standardized and validated data collection instruments and methodology, making the findings more generalizable. In addition, a multilevel-modeling technique was also used in the analysis due to the clustering effect of EDHS data, which provides disaggregated evidence on individual and community-level determinants for designing contextual interventions. To the best of our knowledge, this is the first study to quantify the respective contribution of the individual‑, and community‑level factors for the uptake of health information on NDSs. Despite the aforementioned strengths, the study has limitations due to the cross-sectional nature of the EDHS data. It is impossible to show the cause-effect relationship between the independent variables and the outcome of interest. Recall bias may also exist because study participants were asked about events that occurred two years preceding the survey.

## Conclusion

The findings revealed that unacceptably low uptake of health information on newborn danger signs during the immediate postpartum period in Ethiopia. Wealth index, region, frequency of ANC visit, place of delivery, and receipt of postnatal visits were positively associated with the receipt of health information on NDSs during the immediate postpartum period. In contrast, nulliparity and primiparity were identified as negatively associated with service uptake. Thus, a concerted effort is needed from all stakeholders in the health sector to enhance the uptake of maternal health services (antenatal care, skilled delivery service, and postnatal care). Healthcare providers should pay special attention to nulliparous and primiparous women during and after delivery, and the government should also focus on women who live in peripheral regions, who make up a large portion of the low coverage.

## Data Availability

The data supporting the findings of this study can be obtained in anonymized form from the Demographic and Health Survey website at https://www.dhsprogram.com upon reasonable request in the same way as the authors. The authors did not have any special access privileges that others would not have.

## Abbreviations

AIC: Akaike’s information criterion
ANC: Antenatal Care
AOR: Adjusted odds ratio
COR: Crude odds ratio
CSA: Central Statistical Agency
DHS: Demographic and health survey
EA: Enumeration area
EDHS: Ethiopian Demographic and Health Survey
ICC: Intra Class Correlation Coefficient
NDSs: Neonatal Danger Signs
PCV: Proportional Change in Variance
PNC: Postnatal Care
PPP: Postpartum Period
